# Valve-related complications after mechanical heart valve implantation

**DOI:** 10.1007/s00595-014-1104-0

**Published:** 2014-12-18

**Authors:** Yoshio Misawa

**Affiliations:** Division of Cardiovascular Surgery, Jichi Medical University, 3311-1 Yakushiji, Shimotsuke, Tochigi 329-0498 Japan

**Keywords:** Valve-related complication, Mechanical heart valve, Heart valve replacement, Heart valve surgery

## Abstract

The number of heart valve surgeries is increasing, and 19,164 patients underwent heart valve surgery in Japan in 2011. The early mortality rate has remained stable for more than 10 years. Many patients now survive for many years, with a reported 10-year survival rate of at least 60 %. However, unfavorable complications can occur after valve surgery. Valve-related complications include thromboembolisms, bleeding complications and prosthetic valve endocarditis, followed by structural and nonstructural prosthetic valve dysfunctions. Our review of studies published after 2000 revealed that the rate of all valve-related complications was 0.7–3.5 % per patient-year. Thromboembolisms occur at a rate of approximately 1 % per patient-year, and bleeding complications occur at almost 0.5 % per patient-year. Thromboembolic and hemorrhagic events related to anticoagulant therapy should be considered during life-long follow-up. The occurrence rate of endocarditis reaches 0.5 % per patient-year, with a poor postoperative survival. Structural dysfunctions have been largely overcome, and the nonstructural dysfunction rate is 0.4–1.2 % per patient-year. The nonstructural dysfunctions induced by paravalvular leaks and pannus ingrowth are also issues that need to be resolved.

## Introduction

In 2011, 19,164 patients underwent heart valve surgery in Japan, and the postoperative early death rate was 3.4 % [1]. The number of heart valve surgeries is increasing, and the early mortality rate has remained stable for more than 10 years [2]. Tissue valves are more likely to be implanted than mechanical ones in Japan, with 9,832 tissue and 5,452 mechanical heart valves implanted in 2011. The prolonged durability of the tissue valves, an aging population and the expansion of plastic surgery techniques have all decreased mechanical valve use. However, mechanical valves appear to be a better option for some patients. We choose a mechanical valve for young adults and patients with end-stage renal disease because of the more rapid onset of structural dysfunction of tissue valves [3].

Many patients now survive for many years after valve implantation, with a reported 10-year survival rate of at least 60 % [4–7]. However, unfavorable complications can occur after valve surgery. We herein review and discuss mechanical prosthetic valve-related complications.

## All valve-related complications

Edmunds et al. [8] designed the guidelines for reporting outcomes after prosthetic valve replacement, and many articles have been published based on these guidelines. The authors stated that the valve-related complications include thromboembolisms, bleeding complications and prosthetic valve endocarditis, followed by structural and nonstructural prosthetic valve dysfunctions. Our review of studies published after 2000 revealed a rate of valve-related complications of 0.7–3.5 % per patient-year [2, 8–14]. The occurrence rates of individual complications and references are shown in Table 1.

**Table 1:** The occurrence rates of complications and references

	All valve-related complications	Thromboembolism	Bleeding	Endocarditis	Nonstructural dysfunction
% per patient-year	0.7–3.5	Approximately 1.0	<0.5	<0.5	0.4–1.2
References	[2, 8–14]	[5, 9, 10, 15–19]	[5, 15, 16]	[10, 21–25]	[9, 39–57]

## Thromboembolism and bleeding complications

Thromboembolisms, such as cerebral infarction and prosthetic valve thrombosis, and bleeding complications might be related to the use of warfarin anticoagulation therapy. Other reasons include intrinsic coagulation factors, intestinal lesions, atrial enlargement, arrhythmias such as atrial fibrillation and other causes. A preoperative history of cerebrovascular events is also a risk factor for thromboembolic or bleeding complications [5, 15, 16]. Thromboembolisms occur at a rate of approximately 1 % per patient-year, and bleeding complications at almost 0.5 % per patient-year.

The 2014 American Heart Association/American College of Cardiology guidelines for the management of patients with valvular heart disease state that an international normalized ratio (INR) of 2.5 is recommended in patients with a mechanical valve at the aortic position, and an INR of 3.0 should be obtained for those with a valve implanted at the mitral position [17]. Therapy with 75–100 mg aspirin daily is also recommended. Some authors recommend an INR of 2.6–3.0 for aortic valve replacement patients and 3.0–3.5 for mitral valve replacement patients [9, 10]. The Japanese guideline published in 2012, however, recommended a slightly lower INR between 2.0 and 3.0 [18]. Our present INR target value is between 1.8 and 3.0 for all patients, with additional dipyridamole or aspirin administration [4]. For patients with atrial fibrillation and young patients, our criterion is based on the same recommendation, but for older patients with risk factors for cerebral bleeding, our target INR is below this recommendation.

A thrombosed prosthetic valve is an uncommon complication, but it is associated with high mortality and morbidity rates. In a literature survey of the clinical outcomes of patients with obstructive thrombosed prosthetic heart valves, Huang et al. [19] concluded that thrombolytic therapy was easier than surgery, with a recurrence rate of 13 %, while surgery was necessary in the 30 % of cases with failure of thrombolytic therapy, and was associated with a mortality rate of at least 12 %.

## Endocarditis

Prosthetic valve endocarditis requires complicated surgical procedures [10, 20] and sometimes leads to lethal clinical results, particularly in early-onset patients [21–25]. The occurrence rate of endocarditis is approximately 0.5 % per patient-year. In 1996, Lytle et al. [21] showed that 13 % of 146 patients had in-hospital deaths, and in 2014, Grubitzsch et al. [22] showed that the surgical mortality was 12.8 % among 149 consecutive patients with prosthetic valve endocarditis. Therefore, the surgical risk of prosthetic heart valve endocarditis remains high, and *Staphylococcal* species are the most common causative organisms [20, 21].

The postoperative survival in endocarditis patients is poor. Lytle and colleagues indicated that additional surgery was required in 19 of their patients, with a 60 % survival rate at 10 years among the 127 hospital survivors [2, 4]. Grubitzsch et al. [22] recorded 69 early and late complications, including 35 deaths, 23 recurrences and 11 reoperations among 121 patients with a mean follow-up of 4 years.

## Structural dysfunction

Structural dysfunction in first-generation and initial mechanical valves, such as the Starr-Edwards ball and Björk-Shiley valves, have been reported, although the long-term clinical results of both valves have been excellent [26–32]. The main causes of Starr-Edwards ball valve dysfunction have been ball fracture and cloth wear and tear, leading to valve regurgitation [33, 34]. Björk-Shiley valve dysfunctions include leaflet dislodging and fracture.

These rare complications are typically observed more than three or four decades after implantation. Gunn et al. [31] concluded that the Björk-Shiley valve conferred excellent 30-year survival, was associated with three strut fractures during long-term follow-up, with a freedom from reoperation rate of 91 % at 30 years. Harrison et al. [32] discussed 663 catastrophic failures of the valve among approximately 86,000 patients, and we reported a case of extraction of a worn Björk-Shiley prosthetic valve after 39 years because of the potential risk of structural valve dysfunction [35, 36]. The implanted valve was functioning well in our case, but we identified the worn disc during aortic surgery for an acute aortic dissection. Björk pointed out the potential risk of disc wear and fracture, and the Björk-Shiley valve may be changed to pyrolytic carbon discs [37].

As mentioned above, the structural dysfunction of mechanical valves has been largely resolved, including leaflet dislodging and disc fracture and the current mechanical valves show an extremely low occurrence rate of structural valve dysfunction. Structural dysfunction among major commercially available mechanical valves cannot be recognized, even in children [4–7]. Currently, structural dysfunction remains an issue with tissue valves.

In 2008, Cianciulli et al. [38] reported a case of structural dysfunction in a mechanical prosthetic valve made by Tri-technologies Inc., which is a made-to order manufacturer that produces vales for sale to private customers. This case is a specific example, and not the norm.

## Nonstructural dysfunction

Nonstructural valve dysfunction includes paravalvular leaks without apparent endocarditis, cusp entrapment by a pannus or other causes. Clinically important hemolytic anemia can occur with a lower incidence than these complications [39].

Before the publication of guidelines for a linearized rate for reporting outcomes after prosthetic valve replacement, investigators did not report nonstructural dysfunction systematically. The nonstructural dysfunction rate reported by Edmunds and colleagues was 0.4–1.2 % per patient-year among recent mechanical heart valves [40–43].

Paravalvular leaks without apparent endocarditis and pannus formation often lead to reoperation, and are caused by technical errors, latent prosthetic endocarditis or annular calcification. A minor leak might be subclinical in the aortic position, but hemolysis caused by the leak can lead to reoperation in the mitral position. Akins et al. [44] reported the clinical results of 136 consecutive patients with paravalvular leaks, and primarily repaired or replaced the implanted valves. We reported a case that presented with hemolysis 5 years after a third mitral valve operation [45]. The intraoperative findings revealed severe calcification around the regurgitant orifice, and we successfully repaired the valve primarily. We inferred that the hard calcification and the prosthetic valve ring wore the tissue around the prosthesis. Technical errors should be overcome, and improved histocompatibility of the suture ring of the mechanical valves may also help avoid this complication.

Pannus formation might prevent a leaflet from functioning well, and the presence of a pannus on the outflow of the left ventricle below a mechanical valve could narrow the outflow orifice, causing stenosis. This complication occurs mainly after long procedures. We reported a case with a pannus, in which the pannus was resected using rotatable tilting disc prosthesis, resulting in successful preservation of the intact prosthesis [46]. We also experienced four other reoperation cases due to pannus formation [47, 48]. The earliest case received reoperation at 97 months, and the other three cases were treated between 20 and 39 years after the initial operations. Oh et al. [49] reported a large amount of pannus ingrowth in 33 aortic valve replacement patients with an increased mean pressure gradient. The mean interval from the previous operation was 16.7 ± 4.3 years, and the authors showed that the most common etiology for the previous surgery was rheumatic valve disease. Ellensen et al. [50] also reported a large series of 27 cases, and showed that females and younger patients had a higher risk of pannus formation. Al-Alao et al. [51] reported a rare case with pannus formation 3 months after surgery. Certain intrinsic factors might contribute to pannus formation, and manufacturers have been changing their designs and suture ring fabric compositions to prevent a pannus from developing. The results of these changes will take several decades to become evident.

In addition, diagnosing pannus formation is not easy. The clinical signs of implanted valve dysfunction, such as stenosis, might indicate certain diagnostic procedures, and an echocardiogram is the key to detect valve dysfunction. However, Xu et al. [52] showed that fluoroscopic examination was useful to reveal a radio-opaque ring within the implanted valve orifice, and Teshima et al. [53] studied the effectiveness of multidetector-row-computed tomography to demonstrate pannus overgrowth on the inflow aspect of the prosthetic valve.

Some authors found an annular valve suture interfering with the normal closure mechanism of a prosthetic leaflet, causing valve dysfunction [54, 55]. The normal prosthetic valve function was restored after removing the suture material. Apparent hemolysis without a paravalvular leak has been rarely reported [9, 56, 57]. Borman et al. [57] showed that simultaneous aortic and mitral valve replacement caused relatively increased hemolysis compared with single valve replacements, and indicated that all of six instances treated at their institution did not require reoperation.

## Conclusions

Valve-related complications after heart valve replacement with mechanical valves occur at acceptable rates. The structural dysfunction has been largely overcome; however, prosthetic valve endocarditis may still result in death. Thromboembolic and hemorrhagic events related to anticoagulant therapy should be considered during life-long follow-up. Nonstructural prosthetic valve dysfunctions, such as paravalvular leaks and pannus ingrowth, are also issues that need to be resolved.
